# Suppression of AC railway power-line interference in ECG signals recorded by public access defibrillators

**DOI:** 10.1186/1475-925X-4-65

**Published:** 2005-11-26

**Authors:** Ivan Dotsinsky

**Affiliations:** 1 Center of Biomedical Engineering, Bulgarian Academy of Sciences, Acad. G. Bonchev str., bl. 105, 1113 Sofia, Bulgaria

## Abstract

**Background:**

Public access defibrillators (PADs) are now available for more efficient and rapid treatment of out-of-hospital sudden cardiac arrest. PADs are used normally by untrained people on the streets and in sports centers, airports, and other public areas. Therefore, automated detection of ventricular fibrillation, or its exclusion, is of high importance. A special case exists at railway stations, where electric power-line frequency interference is significant. Many countries, especially in Europe, use 16.7 Hz AC power, which introduces high level frequency-varying interference that may compromise fibrillation detection.

**Method:**

Moving signal averaging is often used for 50/60 Hz interference suppression if its effect on the ECG spectrum has little importance (no morphological analysis is performed). This approach may be also applied to the railway situation, if the interference frequency is continuously detected so as to synchronize the analog-to-digital conversion (ADC) for introducing variable inter-sample intervals. A better solution consists of rated ADC, software frequency measuring, internal irregular re-sampling according to the interference frequency, and a moving average over a constant sample number, followed by regular back re-sampling.

**Results:**

The proposed method leads to a total railway interference cancellation, together with suppression of inherent noise, while the peak amplitudes of some sharp complexes are reduced. This reduction has negligible effect on accurate fibrillation detection.

**Conclusion:**

The method is developed in the MATLAB environment and represents a useful tool for real time railway interference suppression.

## Background

Public access defibrillators (PADs) are now available for more rapid and efficient treatment of out-of-hospital sudden cardiac arrest [1,2]. They are strongly recommended in The American Heart Association and the International Liaison Committee on Resuscitation Guidelines [3]. PADs are used normally by untrained people on the streets, and in theaters, sports centers, airports, and other public arenas. Therefore, automatic fibrillation detection, or its exclusion, is of high importance. Recently [4-6], special attention has been paid to the susceptibility of PADs to the electromagnetic field generated by the train overhead electric power-lines at railway platforms in countries using 16.7 Hz AC power, such as Switzerland, Germany, Austria, Norway and Sweden [4,5,7]. The interference induced may compromise the fibrillation detection [8,9] and increase the risk of inappropriate shock or of not supplying the life-saving defibrillation.

Effective suppression of the railway interference is impeded by its large frequency band. The Official Journal of the European Communities [10] reports possible frequency deviations from 15.69 through 17.36 Hz. No data about the rate of frequency changes are available. Christov and Iliev [6] assume a modulation of 20% around 16.7 Hz per 10 s. There are no papers available on railway interference suppression except for the publication of Christov and Iliev [6] and the recent paper by Jekova and Krasteva [11].

Christov and Iliev [6] apply adaptive filtering based on proposed use of a special antenna within the PAD to feed the reference input of the filter. These authors present good results with simulated experiments of normal ECG signals, tachycardia, and ventricular fibrillation (VF). No signal distortions can be observed. However, the absence of a real embedded antenna is an important disadvantage of the method proposed.

Jekova and Krasteva [11] modify some steps of the subtraction procedure [12,13] for railway interference suppression. The subtraction procedure is known to eliminate 50/60 Hz power-line interference without affecting the QRS spectra. It applies a moving average (comb filter) on linear segments (with frequency band near zero) to remove the interference components, which are stored and further subtracted from the ECG signal wherever non-linear segments are encountered. Jekova and Krasteva [11] band-pass filter the contaminated signal as a preliminary step of a sophisticated approach for linear segment detection inside VF, where these segments, if any, are extremely short. These segments are used for interference frequency measurement, necessary to compensate the fractional part of the inter-sample distance, which appears within a non-rated interference period. The obtained results are generally good. However, the QRS spectrum preservation as a necessary condition for accurate fibrillation detection is contestable. The algorithm is only checked in relatively narrow limits of interference frequency variation: from 16.5 through 17.3 Hz per 10 s. Linear segment detection is applied to signals with normal ECG activity, where no difficulties are expected; and in epochs with fibrillation, where compromises with the detection accuracy have no visible effect because of the low frequency spectra of the waves. No example with transition from normal activity to fibrillation is presented. The resulting amplitude errors are calculated by averaging the distortions over 1 s.

## Method

### Materials

The study was carried out in the MATLAB environment. ECG recordings were taken from the AHA database, section 8. They contain normal cardiac rhythm followed by fibrillation. The sampling rate (SR) is 250 Hz. Sinusoidal oscillations within the band of the railway interference were synthesized and added to the ECG signals. The mixed signals were subjected to some filtrations and digital procedures for interference suppression. The results obtained are also valid for the harmonics and, therefore, may be extrapolate for any arbitrary waveform. The interference suppression as well as the differences between input and processed signals were assessed. The algorithm and program developed have a structure that simulates a real-time ongoing procedure.

### Assessment of some traditional filtrations applied for railway interference suppression

Railway power-line interference may be suppressed by an appropriate notch filter, a moving average (comb filtering with linear phase characteristic), or other relatively simple techniques. All such procedures have the common disadvantage of affecting the ECG spectrum, especially to reduce the amplitude mostly of high and steep QRS complexes. However, it is very unlikely that such shape alternation would cause failure of the algorithms for fibrillation detection. The reason is that the variety of ECG signals is immense, including relatively low frequency low amplitude QRS complexes. Consequently, each processed signal may be assumed to be equal or very close to some non-processed signal. This statement will be supported below by the signals illustrating the proposed approach for 16.67 Hz interference suppression.

Therefore, only the level of the residual interference is further assessed and taken into consideration as a limitation of fibrillation detection.

**Figure 1:**
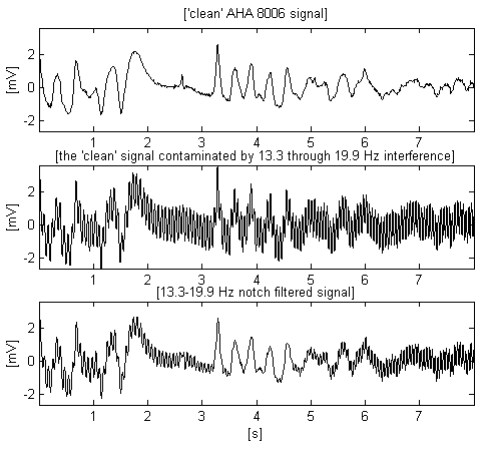
Band-stop filtering of contaminated ECG signal.

The first trace of Fig. 1 shows an 8 s epoch of the AHA 8006 signal. It is assumed to be a 'clean' input signal, which is further mixed with interference of ± 1 mV amplitude and variable frequency from 13.3 trough 19.9 Hz (second trace). As can be seen, the residual interference after notch filtration may compromise the fibrillation detection. No difference between input and processed signal is shown here, as obviously it is too large to be compared with the differences obtained by other approaches.

**Figure 2:**
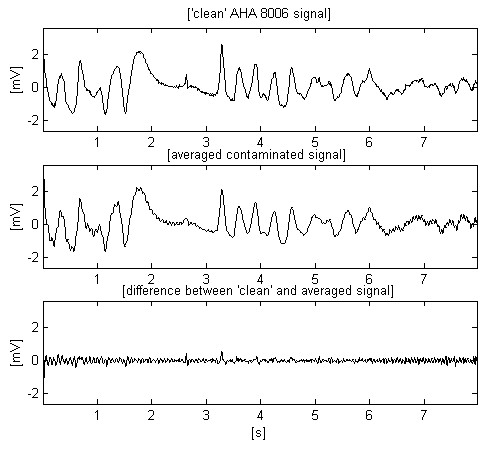
Moving averaging (comb filtering) of the same contaminated ECG signal.



The same input signal is used for estimation of simple moving averaging (Fig. 2). For odd sample number, *n *= *2m *+ *1 *in one period of the interference, the ongoing middle filtered sample *Y*_*i *_is given by:

Here *X*_*i*+*j *_stands for the surrounding non-filtered samples. The processed signal (second trace) shows improved interference suppression mainly in the middle part of the epoch where the interference frequency coincides with the first zero of the comb filter. The third trace demonstrates partially eliminated interference together with clipped peaks of some sharp complexes.

Adaptive and non-adaptive filters [14,15] are not considered because of their unacceptably long transient times appearing every time the ECG signal course changes.

### Proposed approach

The second trace of Fig. 2 suggests that good suppression can be obtained if the first comb filter zero follows the interference frequency change. As the number of averaged values is integer, the inter-sample intervals have to be modified by adaptive SR of the analog-to-digital conversion (ADC). This approach has been developed as a part of the subtraction procedure [12,13] and implemented by hardware measuring of the interference frequency. However, this technique is not suitable for battery-supplied devices and computer-aided systems.

**Figure 3:**
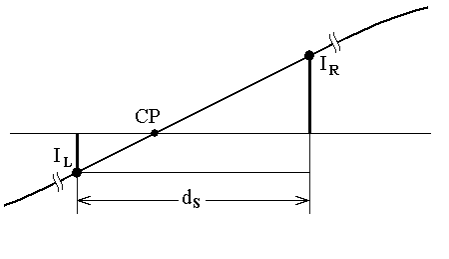
Software measuring of the interference frequency.

Recently, Dotsinsky and Stoyanov published an alternative software method [16]. The contaminated signal is digitally band-pass filtered at -3 dB from 49 through 51 Hz. The amplitudes of two adjacent samples on a positive-going slope of the interference signal, located below and above the zero line, are measured. Then, the crossing point, *CP*, of the interference with the zero line is determined by interpolation using the two homogenous triangles defined by the left and right interference samples *I*_*L *_and *I*_*R *_(Fig. 3). The location of *CP *on the inter-sample distance *d*_*S *_is used to calculate the ongoing fluctuation of the interference period of repetition.

Although the adaptation of the inter-sample distances does not exceed the limit of 1% for 50/60 Hz interference, another solution proposed to eliminate the adaptive ADC, is preferred [17]. The rated ADC is applied without any adjustment. A moving averaging over a constant number of samples, *n*, is carried out. This average defines total suppression of interference with rated frequency. The ongoing frequency is software measured, and based on the result, an internal re-sampling is carried out. Linear interpolation is used to create new sample values with irregular inter-sample distances in contrast to the digitized input ECG signal but regularly spaced towards the rated interference frequency. Therefore, the interference is suppressed regardless of its original frequency. Then, the 'clean' ECG signal obtained is subjected to back re-sampling, restoring the rated (regular) inter-sample distances. Linear interpolation is used once more.

This method is modified for railway interference cancellation to overcome its inherent large and fast frequency variations. Another difficulty derives from the almost total overlap between the spectra of the QRS complexes and the interference, which reduces the accuracy of the linear interpretation.

The contaminated signal is processed by a first order Butterworth band-pass filter. When *SR *= 250 Hz, the recursive equation used is:

*Y*_*n *_= *1.7168Y*_*n-1 *_*- 0.8529Y*_*n-2 *_*+ 0.0735(X*_*n *_*- X*_*n-2*_*)*,     (2)

which defines the lower cut-off at 12.5 Hz and the upper cut-off at 18.8 Hz. *Y*_*n *_and *X*_*n *_are the ongoing filtered and non-filtered samples, respectively. The other variables represent previous filtered and non-filtered samples. Furthermore, *n *is chosen equal to 15 so as to set the first zero of the comb filter at 16.67 Hz.

**Figure 4:**
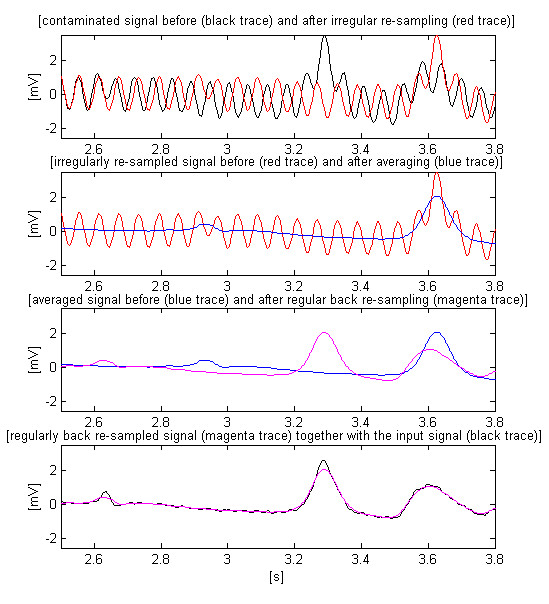
Four steps of the two-way re-sampling.

The four steps of the two-way re-sampling are illustrated in Fig. 4. For a better observation, the signal is limited inside 2.5 and 3.8 s.

**Figure 5:**
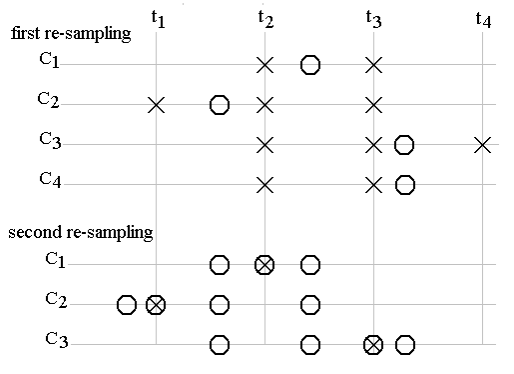
Possible cases of interpolation.

Both linear interpolations seem to be simple procedures. However, since the program written in the MATLAB language simulates a real-time ongoing process, each cycle begins with ongoing sample of the input signal, and the sequence of non-alternative forward and back interpolations requires a more sophisticated coordination of these steps. The possible cases are presented in Fig. 5. A pointer *P*_*r *_is assigned to the regular ADC positions marked by 'X'. Two other pointers, *P*_*f *_and *P*_*s*_, control the locations 'O' and '' for first and second re-sampling, respectively. All pointers are incremented at the beginning of the cycles.

The upper part of Fig. 5 presents four possible cases of the first re-sampling C_1_-C_4_. C_1 _is the simplest case of interpolation. *P*_*r *_indicates the position t_3_. The sample to be calculated, 'O', is inside the two top ADC samples of *P*_*r *_located at t_2 _and t_3_. Its value depends on the two top ADC samples amplitudes and their distance from 'O'. If the last 'O' address of *P*_*f *_(case C_2_) is on the left of the interval defined by two consecutive top 'X' addresses of *P*_*r *_(case C_2_, t_2_and t_3_), this pointer is decremented (the two 'X' addresses are shifted at t_1 _and t_2_) before the interpolation is accomplished in the next cycle (when all pointers will be incremented). Furthermore, C_3 _and C_4 _show the last *P*_*f *_address on the right of the interval t_2_-t_3_. *P*_*r *_is incremented before the interpolation in the current cycle, if possible (case C_3 _with subsisting ADC sample at t_4_). Otherwise, in case of C_4_, *P*_*f *_is decremented without interpolation to hold the address inside the interval in the next cycle.

The second re-sampling, shown in the lower part of Fig. 5, follows the same rules of the first re-sampling (cases C_1_-C_3_), except for the last case, C_4_. Here the procedure is unrealistic because the program begins after a certain delay of *P*_*f *_and *P*_*s *_is reached in respect to *P*_*r*_.

**Figure 6:**
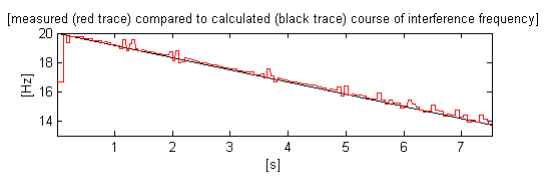
Comparison between measured and preliminary calculated course of the synthesized interference.

In contrast to the software measurement of 50/60 Hz interference [16,17], the influence of wide and high QRS complexes in case of 16.67 Hz interference is an order of magnitude higher. This influence is reduced by clipping the calculated inter-sample intervals beyond a level as defined by a percentage of the weighted sum of the two last intervals. Thus the measured values are restricted to be within expected possible fluctuations, which are better related to the current interference frequency. A constant clipping level will introduce erroneously different allowed rates of frequency changes for the ranges around 13 and 19 Hz. The efficiency of the approach can be seen in Fig. 6, where the black trace represents the preliminary calculated course of the synthesized interference while the red trace is for the measured frequency.

In summary, the proposed approach consists of rated ADC, software frequency measuring, internal irregular re-sampling according to the interference frequency, and moving averaging over a constant number of samples followed by regular back re-sampling.

## Results

The features of the developed algorithm may be assessed in the following figures, where no residual noise in the processed signal can be seen.

**Figure 7:**
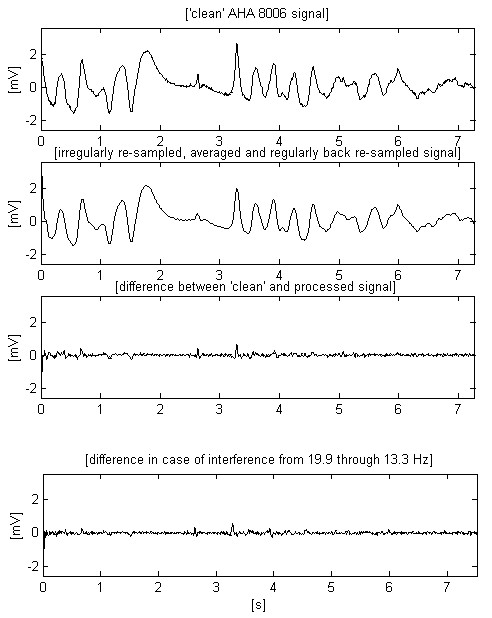
Result of applying the proposed method on the ECG signal of Figs. 1 and 2.

The second trace of Fig. 7 shows a total railway interference cancellation together with suppression of inherent signal noise, e.g., muscle disturbances inherent to the 'clean' input signal. The two last traces prove the independence of the method with respect to the direction of the interference frequency change.

**Figure 8:**
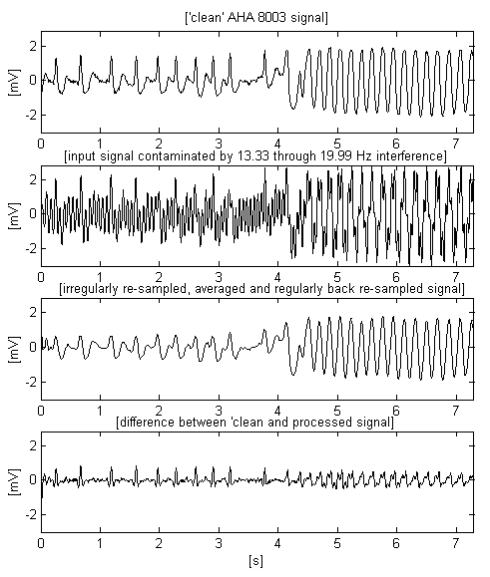
Processing of signal, which presents transition from normal heart activity to fibrillation.

**Figure 9:**
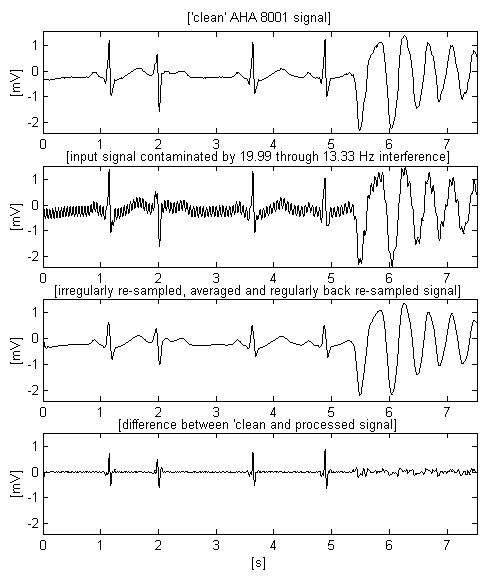
High and steep QRS complexes superimposed by interference with lower amplitude, which is the worst case of using the software frequency measurement.

**Figure 10:**
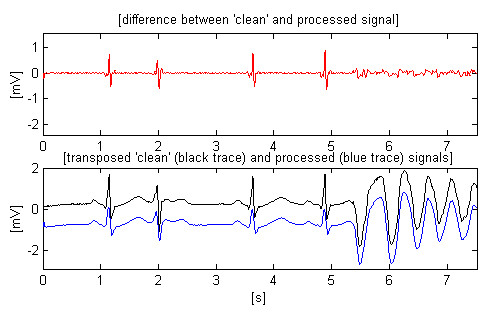
Effect of the slight shifting of the processed signals because of the high dynamics of the amplitude change that leads to irregular group delay after the band-pass filtering.

Two other AHA signals, chosen to demonstrate the transition from normal heart activity to fibrillation, can be observed in Figs. 8 and 9. The second signal includes high and steep QRS complexes, and has superimposed interference with lower amplitude, which is the worst case of using software frequency measurement of interference [17]. The shown differences between input and processed sharp complexes are to some extent due to the slight shifting within the processed signals. It is a consequence of the irregular transition time (group delay), introduced by the band-pass filtering of signal parts with relatively rapid amplitude change. This effect may be better seen in Fig. 10, where the first trace represents the difference between input and processed signals of Fig. 7, while the second trace points out the delay introduced between them.

**Figure 11:**
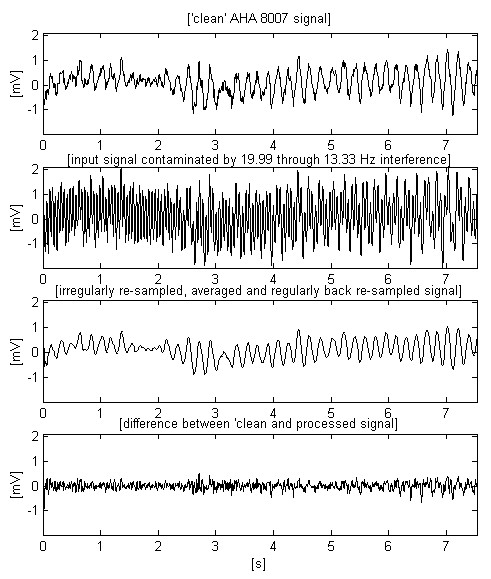
Epoch extracted from the AHA 8007 signal with higher frequency spectrum and smaller amplitude.

**Figure 12:**
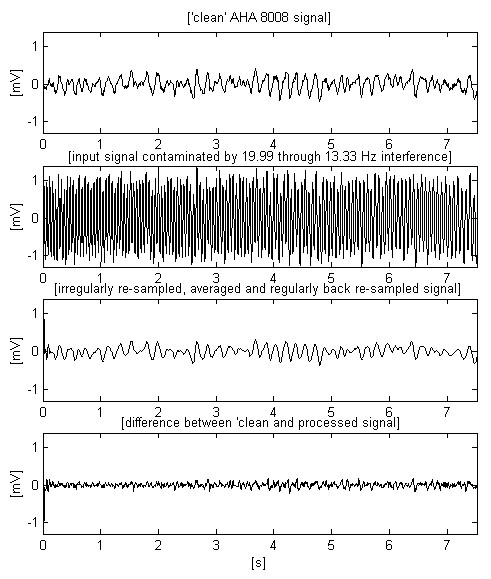
Epoch extracted from the AHA 8008 signal with higher frequency spectrum and smaller amplitude.

The signals presented in Fig. 11 and Fig. 12 are chosen to test the method with VF, which has a higher frequency spectrum and smaller amplitude.

Let us compare the two first traces in Fig. 7. If the non-filtered signal has a given probability for correct VF detection, than this probability must be the same for the filtered signal (second trace) despite the smoothed peak immediately after the 3^rd ^s. In the present study, this statement may be only visually supported by knowledge and experience in the analysis of ECG signals. The alternative approach consists of testing a representative set of fibrillation detection algorithms on various ECG signals superimposed by 16.67 Hz interference. However, such a study is out of the aim of this paper. Besides, it is very time-consuming because of the enormous volume of experiments needed for a good statistic. The same considerations may be supported looking at Fig. 9. The filtered third trace has preserved sufficient parameter differences between QRS complexes and fibrillation waves for a correct detection of transition to fibrillation. These signals, as well others tested but not shown here, confirm the conviction that the moving averaging does not compromise the detection of fibrillation.

## Discussion and conclusion

Our algorithm developed in the MATLAB environment is a useful tool for real-time railway interference suppression. The time required for off-line analysis of an 8 s epoch with the program, structured to simulate a real-time going process, is less than 4 s. The shape deviations for high and steep QRS complexes are assumed to be negligible for accurate fibrillation detection. These deviations are due to the moving averaging and suppressed noise inherent to the 'clean' signals taken from AHA database. The real differences between 'clean' and processed contaminated signals are smaller than those shown in the Figures, where the non-identical group delay of the band-pass filtering results in shifting the signals relative to each other. It may be possible that the real differences can be further reduced if 4^th ^or 8^th ^point Lagrange interpolation is used. This possibility is tested with 50/60 Hz interference [17], where it is found to have insignificant increase on the accuracy, perhaps because of the relatively narrow range of interference frequency fluctuations.
